# Evaluation of the predictive accuracy of nine staging systems in patients with hepatocellular carcinoma: a multicenter, retrospective study in China

**DOI:** 10.1038/s41598-026-50816-4

**Published:** 2026-06-04

**Authors:** Shibo Wang, Yu Li, Shuang Leng, Chao Li, Hong Zhu, Jing Lv, Dayong Zheng, Haifeng Lin, Jianhong Zhong, Ming Zhao, Jun Xue, Peng Huang, Zhiyu Chen, Qian Zhu, Weijia Fang, Xiufeng Liu, Yan Yang, Nanya Wang

**Affiliations:** 1 Department of Phase I Clinical Trial Center, The First Hospital of Jilin University, Changchun, Jilin China; 2 Department of Medical Oncology, Hongqi Hospital, Mudanjiang Medical University, Mudanjiang, Heilongjiang China; 3 Department of Hematology and Oncology, Meihekou Central Hospital, Meihekou, Jilin China; 4 Department of Hepatobiliary Surgery, Eastern Hepatobiliary Surgery Hospital, Second Military Medical University, Shanghai, China; 5 Cancer Center, The First Affiliated Hospital of Soochow University, Soochow, Jiangsu China; 6 Department of Medical Oncology, The First Affiliated Hospital of Qingdao University, Qingdao, Shandong China; 7 Department of Oncology, Nanfang Hospital, Southern Medical University, Guangzhou, Guangdong China; 8 Department of Medical Oncology, The Second Affiliated Hospital of Hainan Medical College, Haikou, Hainan China; 9 Hepatobiliary Surgery Department, Guangxi Medical University Cancer Hospital, Nanning, Guangxi China; 10 Department of Oncology, Cancer Center, The First Affiliated Hospital, Sun Yat-sen University, Guangzhou, Guangdong China; 11 Department of Surgery, Wuhan Union Hospital, Wuhan, Hubei China; 12 Department of Gastroenterology, Harbin Medical University Cancer Hospital, Harbin, Heilongjiang China; 13 Institute of Hepatobiliary Surgery, The Southwest Hospital of Army Medical University, Chongqing, China; 14 Department of Institute of Hepatobiliary Diseases, the Zhongnan Hospital of Wuhan University, Wuhan, Hubei China; 15 Cancer Biotherapy Center, First Affiliated Hospital, Zhejiang University, Hangzhou, Zhejiang China; 16 Jinling Hospital, Nanjing University, Nanjing, Jiangsu China; 17 Department of Medical Oncology, The First Affiliated Hospital of Bengbu Medical University, Bengbu, Anhui China

**Keywords:** Liver cancer, Therapeutic approaches, Staging system, Prognostic evaluation, Cancer, Gastroenterology, Oncology

## Abstract

There are several staging systems for hepatocellular carcinoma (HCC); however, the best prognostic system for Chinese patients has not been established. The aim of this study was to evaluate nine staging systems and identify the best prognostic staging system for Chinese patients with HCC. This retrospective study included 1,495 patients with HCC from 2012 to 2022 in 17 hospitals in China. The predictive ability of nine HCC staging systems was evaluated based on homogeneity, monotonicity of gradients, Akaike’s information criterion, and area under the receiver operating characteristic curve. Overall survival at 1, 3 and 5 years was 59.90%, 40.34%, and 34.83%, respectively. Multivariable Cox analysis identified older age, multiple tumor nodules, maximum tumor size > 5 cm, macrovascular invasion, distant metastases, treatment modalities, worse performance status, low protein, high bilirubin, and high alpha-fetoprotein as independent prognostic factors. The Hong Kong Liver Cancer (HKLC) system showed the highest predictive accuracy for Chinese patients in hepatitis B virus-infected populations and in those undergoing non-curative treatment. The Cancer of the Liver Italian Program showed the best predictive ability in patients with hepatitis C virus infection or in the curative treatment subgroup. HKLC may be the most consistent and reliable prognostic model for patients with HCC in the Chinese population.

## Introduction

Hepatocellular carcinoma (HCC) is the fourth most common cancer globally and the second leading cause of cancer-related mortality^1–3^. According to the 2022 Global Cancer Statistics report, approximately 45% of new HCC cases and 47% of HCC deaths occur in China^1^. Recent literature estimated the occurrence of 431,000 new HCC cases in China by 2025^1,4^. The majority of HCC cases in China are strongly associated with hepatitis B virus (HBV) infection, which contrasts with Western countries, where HCC is primarily linked to hepatitis C virus (HCV) infection, alcoholic liver disease, and metabolic-associated fatty liver disease (MAFLD)^5,6^. Therefore, given that the development and progression of HCC are strongly influenced by the underlying etiology, the optimal staging system for HCC may vary between regions depending on the predominant etiological factors^7^.

Unlike other solid malignancies, the prognosis of liver cancer is influenced not only by the biological characteristics of the tumor but also by the functional reserve of the liver^8,9^. Therefore, evaluating the prognosis of patients with HCC requires a comprehensive assessment of liver function, tumor extent, general health status, and treatment strategy. The BCLC staging system, endorsed by both the American Association for the Study of Liver Diseases (AASLD) and the European Association for the Study of the Liver (EASL), has become an important tool for standardizing staging and treatment strategies^10–12^. This system integrates crucial indicators such as liver function and tumor extent, providing treatment recommendations based on disease stage. However, as the BCLC system was developed primarily for HCV-related liver disease in Western populations, its applicability to the Chinese population, which is predominantly affected by HBV, is limited^13^. In response, the 2017 Chinese Guidelines for the Diagnosis and Treatment of Primary Liver Cancer incorporates the China Liver Cancer Staging (CNLC) system, designed to account for the specific liver disease characteristics of Chinese patients and enable more accurate, individualized treatment^14^.

Artificial intelligence (AI) has advanced rapidly in recent years, and efforts have been made to integrate AI with traditional staging methods to improve prognostic prediction^15–17^. However, the complex computational models and high technical barriers associated with AI limit its clinical applicability, while traditional staging systems based on clinical features and laboratory indicators remain the primary tools in clinical practice^18^. Since the CNLC staging system was incorporated into Chinese HCC treatment guidelines in 2017, several studies have evaluated the prognostic efficacy of various staging systems (e.g., BCLC, TNM, JIS) in Chinese patients with HCC^19–21^. However, most of these studies are limited to single analyses or efficacy verifications of specific treatment modalities. For example, studies on patients undergoing transarterial chemoembolization (TACE), the CNLC staging system has consistently demonstrated the best predictive power^20^. In contrast, other studies focusing on unresectable HCC in China have shown that CUPI provides the best predictive power, while CLIP has been the least effective^21^. These conflicting findings highlight the ongoing controversy regarding the optimal staging system for HCC in China.

Therefore, this study aims to systematically evaluate, for the first time, the prognostic efficacy of nine staging systems—CNLC, BCLC, GRETCH, JIS, HKLC, CUPI, Okuda, Tokyo, CLIP—in a Chinese population with HCC. The goal is to provide critical theoretical and clinical evidence to support the establishment of a consensus staging system tailored to the specific characteristics of Chinese individuals with liver cancer.

## Patients and methods

### Patients

Patients with newly diagnosed HCC between January 2012 and December 2022 were included. Patients with recurrent HCC, synchronous or previous other primary malignancies, or incomplete follow-up data were excluded. Professional staff collected comprehensive baseline data, including demographic characteristics, laboratory biomarkers, tumor-related imaging findings, and physical performance scores, in accordance with a standardized protocol uniformly implemented across all participating centers. Patients were monitored every 3 to 6 months until study completion, death, or loss to follow-up. The study protocol was approved by the medical ethics committee of The First Hospital of Jilin University (No. 2026-026). Informed consent was obtained from all participants after they had been fully informed about the study prior to their participation. Patient anonymity was maintained by excluding personal identifiers before analysis. The study adhered to the Declaration of Helsinki and the Ethical Guidelines for Clinical Research throughout all stages of data analysis.

### Diagnosis and follow-up

This study followed Chinese guidelines for diagnosing and treating HCC, utilizing enhanced computed tomography (CT), magnetic resonance imaging (MRI), or pathological biopsy for confirmed diagnosis^2,22^. The nine staging systems were retrospectively applied to perform staging assessment for all enrolled patients. Personalized anti-cancer treatment was tailored based on tumor staging, patient physical condition, and liver function, referencing the BCLC (pre-2017) or CNLC (post-2017) guidelines^14^. Patients underwent periodic follow-up assessments at the outpatient facilities of each participating hospital, and the follow-up strategy was consistent in all the participating hospitals. The follow-up protocol included history taking, physical examination, serum alpha-fetoprotein (AFP) assessment, and abdominal imaging via ultrasonography, contrast-enhanced CT, or MRI. This routine was conducted every 2 to 3 months for the first 6 months post-therapy, every 3 to 4 months for the subsequent 18 months, and then every 3 to 6 months thereafter. Patients were considered lost to follow-up if they had no documented clinical encounters for more than six consecutive months or could not be reached by telephone or other communication methods. Tumor recurrence was identified by the emergence of new intrahepatic or extrahepatic nodules, with or without elevated serum AFP levels, displaying typical HCC imaging characteristics on enhanced CT or MRI. Tumor progression was evaluated according to the Response Evaluation Criteria in Solid Tumors (RECIST) version 1.1, defined as at least a 20% increase in the sum of diameters of target lesions or the appearance of new lesions. All imaging findings were reviewed by board-certified radiologists at each participating center^23^.

### Statistical analysis

Statistical analyses were conducted using R v4.3.2 (R Foundation for Statistical Computing) and IBM SPSS Statistics v26.0. Overall survival (OS) was defined as the time from initial diagnosis (enrollment period: January 2012 to December 2022) to death from any cause. Patients who were alive at the last follow-up or lost to follow-up were treated as censored observations at the date of last contact no later than the final follow-up cutoff of December 31, 2022. Continuous variables are expressed as median and interquartile range (IQR), and patients were dichotomized based on the median. Categorical variables are presented as counts (n) and percentages (%). Variables with missing data that may have affected prognosis were uniformly handled using stratified median imputation. Cox proportional hazards regression models were used to identify independent risk factors influencing patient prognosis. Survival curves for all patients were generated using the Kaplan–Meier method. Log-rank tests assessed the significance of survival curve separation. The efficacy of a staging system in predicting HCC prognosis depends on three key factors: (1) Homogeneity, which is the system’s capacity to categorize patients with analogous survival outcomes within the same stage, thereby minimizing variability; (2) Monotonicity, which describes the consistent relationship between the stages and survival rates, ensuring that patients in earlier stages have better survival outcomes than those in more advanced stages; and (3) Discriminatory ability, which refers to the system’s capacity to clearly distinguish between stages, demonstrating significant survival differences among patients at different stages^24^. The prognostic performance of the nine HCC staging systems was assessed using Cox proportional hazards regression analysis. The likelihood ratio χ² (LHR χ²) test was used to evaluate the homogeneity within each staging system and its ability to discriminate survival among different stages. The linear trend χ² test was used to assess the monotonicity of the survival gradient across ordered stages. Higher LHR χ² and linear trend χ² values indicated better prognostic stratification^25^. The discriminatory ability of each staging system in predicting 1-, 3-, and 5-year mortality was evaluated using the area under the receiver operating characteristic curve (AUC). The Akaike Information Criterion (AIC) was further used to compare the overall goodness-of-fit of the models, with a lower AIC value indicating a better-performing staging system. All *P*-values were calculated using two-tailed tests, with *P* < 0.05 considered statistically significant.

## Results

### Study cohort characteristics

**Fig. 1:**
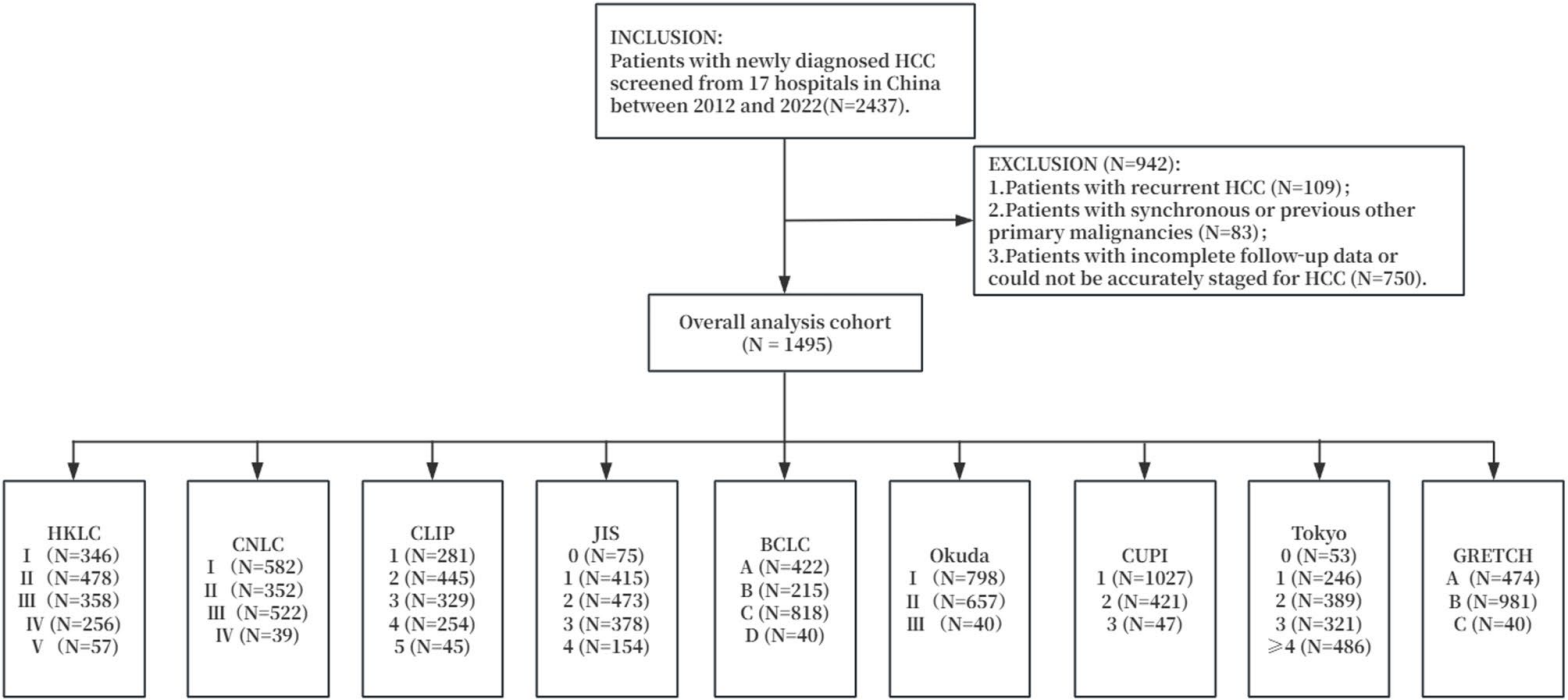
Flow chart of patient selection and numbers of patients in each stage of the nine hepatocellular carcinoma staging systems. HCC, hepatocellular carcinoma; HKLC, Hong Kong Liver Cancer; BCLC, Barcelona Clinic Liver Cancer; CLIP, Cancer of Liver Italian Program; JIS, Japan Integrated Staging score; CNLC, China liver cancer staging; CUPI, Chinese University Prognostic Index; GRETCH, Groupe d’Etude et de Traitement du Carcinome Hépatocellulaire.

Figure 1. shows the patient selection flow chart and stage-specific patient numbers across the nine HCC staging systems. The study included a total of 1,495 patients, with a median age of 58 years. Males comprised 85.8% of the cohort, while females made up 14.2%. Almost half of the patients (47%) had tumors larger than 5 cm, and 54.2% had multiple tumor lesions. Chronic liver disease was attributed solely to HBV infection in 76.5% (1,144) and to HCV infection in 3.9% (58) of the patients. Macrovascular invasion was observed in 508 (34.0%) patients, and extrahepatic metastases were found in 18.7% of patients. Six hundred and forty-six (646) patients (43.2%) received curative treatment (resection, ablation, or liver transplantation), while the remaining 56.8% did not receive curative treatment. More detailed information about the baseline profile of the patients is provided in Table 1. During the median OS of 15.4 months, 551 (36.9%) deaths occurred. The 1-year, 3-year, and 5-year survival rates were 59.90%, 40.34%, and 34.83%, respectively.

**Table 1:** Clinical characteristics of all patients.

Characteristics	All Patients (*N* = 1495)
Age (IQR)	58 (49–65)
Male sex (%)	1282 (85.8)
ECOG score (%)	
0	831 (55.6)
1	634 (42.4)
2	26 (1.7)
> 2	4 (0.3)
Diabetes mellitus (%)	212 (14.2)
HBV (+) (%)	1144 (76.5)
HCV (+) (%)	58 (3.9)
Cirrhosis (%)	1032 (69.0)
Portal hypertension (%)	508 (34.0)
Largest tumor size > 5.0 cm (%)	703 (47.0)
Multiple tumors (%)	810 (54.2)
Extrahepatic spread (%)	279 (18.7)
Macrovascular invasion (%)	377 (24.8)
Blirubin (U/L) (IQR)	17.5 (12.7–24.4)
Albumin (U/L) (IQR)	38.9 (35.0-42.5)
ALT level (U/L) (IQR)	35.2 (23.0–56.0)
AST level (U/L) (IQR)	42.0 (29.0–68.0)
AFP level (U/L) (IQR)	113.3 (8.0-1200.0)
PLT (*10^9/L) (IQR)	147.0 (101.0-204.0)
INR (IQR)	1.03 (0.96–1.12)
Treatment modality (%)	
Curative treatment	646 (43.2)
Resection	486 (32.5)
Ablation	156 (10.4)
Liver transplantation	4 (0.3)
Non-curative treatment	849 (56.8)
TACE	504 (33.7)
Radiotherapy	9 (0.6)
Systemic therapy	301 (17.9)
Palliative treatment	35 (2.3)

**Abbreviations**: ECOG, Eastern Cooperative Oncology Group; HBV, viral hepatitis type B; HCV, viral hepatitis type C; IQR, interquartile range; ALT, Alanine aminotransferase; AST, Aspartate aminotransferase; AFP, alpha-fetoprotein; PLT, blood platelet; PT, Prothrombin time; TACE, transarterial chemoembolization.

### Baseline predictors of survival

Among possible predictors of survival, only baseline total bilirubin had missing data in 61 patients (4.1%), which were imputed by subgroup median values stratified by Child-Pugh grade. All other possible predictors of survival were complete without missing data. Univariate Cox analysis identified several baseline variables associated with survival in patients with HCC, including low albumin, high bilirubin level, high AFP level, tumor multi-nodularity, larger tumor diameter, macrovascular invasion, extrahepatic metastases, poorer ECOG scores, and receipt of non-curative treatments (Table 2, P -values of all factors were less than 0.05). Factors with *P*-values < 0.1 from univariate analysis were included in the Cox multivariable survival analysis.

Multivariable Cox analysis revealed significant risk factors: albumin < 39 U/L (hazard ratio (HR): 1.389, *P* < 0.001, 95% confidence interval (CI): 1.163–1.658), bilirubin > 18 U/L (HR: 1. 366, *P* < 0.001, 95% CI: 1.152–1.621), AFP level > 113 µg/L (HR: 1. 491, *P* < 0.001, 95% CI: 1.248–1.781), maximal tumor size > 5 cm (HR: 1.727, *P* < 0.001, 95% CI: 1.439–2.074), tumor number (HR: 1. 427, *P* < 0.001, 95% CI: 1.187–1.715), macrovascular invasion (HR: 2.105, *P* < 0.001, 95% CI: 1.740–2.547), ECOG (HR: 1.675, *P* < 0.001, 95% CI: 1.440–1. 900), extrahepatic dissemination (HR: 2.578, *P* < 0.001, and 95% CI: 2.089–3.181), and curative treatment (HR: 0.724, *P* < 0.001, 95% CI: 0.589–0.889) were independent predictors of poor prognosis.

**Table 2:** Variables with prognostic value at univariate and multivariable analysis in 1,495 patients with HCC.

Variables	Univariate analysis			Multivariable analysis		
	Risk ratio	*P*-value	95% CI	Risk ratio	*P*-value	95% CI
Age > 58 yrs	1.146	0.134	0.959–1.369			
Albumin < 39 U/L	1.282	0.012	1.056–1.547	1.389	< 0.001	1.163–1.658
ALT level > 35 U/L	0.990	0.956	0.809–1.222			
AST level > 42 U/L	1.154	0.230	0.917–1.435			
Bilirubin > 18U/L	1.276	0.011	1.056–1.518	1.366	< 0.001	1.152–1.621
AFP level > 113 µg /L	1.480	< 0.001	1.233–1.778	1.491	< 0.001	1.248–1.781
INR of PT > 1	1.078	0.420	0.897–1.294			
Platelet < 150,000/µl	0.936	0.476	0.770–1.129			
Largest tumor size > 5 cm	1.732	< 0.001	1.427–2.088	1.727	< 0.001	1.439–2.074
Tumor number	1.453	< 0.001	1.202–1.744	1.427	< 0.001	1.187–1.715
Macrovascular invasion	2.039	< 0.001	2.121–3.266	2.105	< 0.001	1.740–2.547
HBV (+)	1.000	0.962	0.863–1.233			
HCV (+)	0.933	0.767	0.587–1.481			
ECOG	1.601	< 0.001	1.392–1.848	1.675	< 0.001	1.460–1.921
Cirrhosis	0.899	0.293	0.726–1.101			
Portal-hypertension	1.195	0.111	0.961–1.474			
Ascites	1.141	0.125	0.966–1.333			
Extrahepatic spread	2.631	< 0.001	2.121–3.266	2.578	< 0.001	2.089–3.181
Drinking	1.129	0.056	0.998–1.275	/	0.486	/
Smoking	0.887	0.086	0.774–1.017	/	0.626	/
Diabetes	1.040	0.087	0.994–1.089	/	0.087	/
Curative treatment	0.787	0.022	0.625–0.964	0.724	0.002	0.589–0.889

**Abbreviations**: ECOG, Eastern Cooperative Oncology Group; HBV, viral hepatitis type B; HCV, viral hepatitis type C; IQR, interquartile range; ALT, Alanine aminotransferase; AST, Aspartate aminotransferase; AFP, alpha-fetoprotein; CI, confidence interval.

### Survival and stratification based on the nine staging systems

**Fig. 2:**
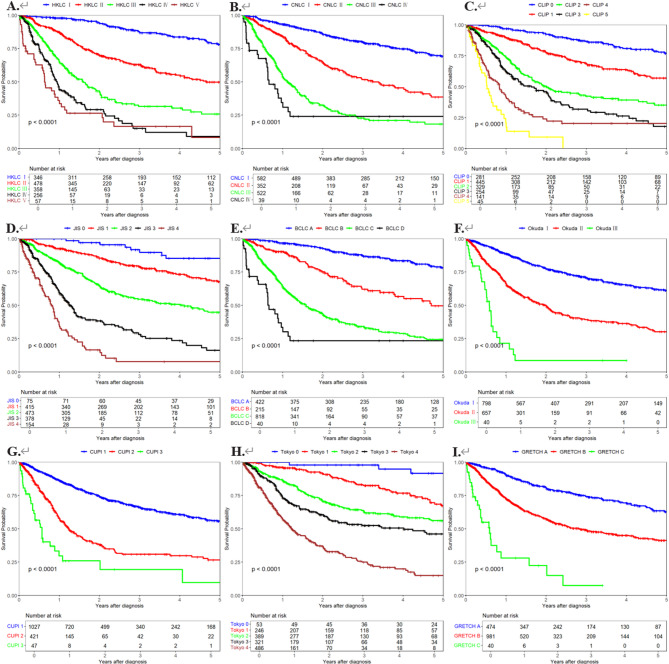
Kaplan–Meier estimated survival curves of all the 1,495 patients diagnosed with hepatocellular carcinoma stratified by (**A**) HKLC, (**B**) CNLC, (**C**) CLIP, (**D**) JIS, (**E**) BCLC, (**F**) Okuda, (**G**) CUPI, (**H**) Tokyo, (**I**) GRETCH. HKLC, Hong Kong Liver Cancer; BCLC, Barcelona Clinic Liver Cancer; CLIP, Cancer of Liver Italian Program; JIS, Japan Integrated Staging score; CNLC, China liver cancer staging; CUPI, Chinese University Prognostic Index; GRETCH, Groupe d’Etude et de Traitement du Carcinome Hépatocellulaire.

Patients were classified according to each of the nine staging systems. The proportions of patients in each stage were determined. In addition, the corresponding cumulative survival rates at 1, 3, and 5 years are shown in Table 3. The distributions of survival, presented as Kaplan–Meier curves (Fig. 2A and I), illustrate the survival across the different staging classifications within each system. Log-rank tests confirmed significant differences in OS rates between certain stages across all nine staging systems (*P* < 0.05), except for stages IV and V of the HKLC system and stages Ⅲ and Ⅳ of the CNLC system.

**Table 3:** Patient distribution and estimated survival rates according to the nine staging systems.

Staging system	No. of patients	Median survival (months)	1-year survival (%)	3-year survival (%)	5-year survival (%)	*P*-value among all stages	*P*-value between two adjacent stages
HKLC							
Ⅰ	346	42.1	97.9	87.2	78.1	< 0.001	
Ⅱ	478	21.7	87.6	63.0	49.9		Ⅰ vs. Ⅱ < 0.001
Ⅲ	358	9.9	65.3	31.6	25.9		Ⅱ vs. Ⅲ < 0.001
Ⅳ	256	6.1	44.1	15.1	9.0		Ⅲ vs. IV < 0.001
V	57	6.9	33.7	16.5	8.3		IV vs. Ⅴ 0.085
BCLC							
1	422	40.9	96.8	87.2	78.3	< 0.001	
2	215	19.4	90.4	62.1	49.6		1 vs. 2 < 0.001
3	818	9.9		34.5	22.2		2 vs. 3 < 0.001
4	40	7.2	45.6	31.5	31.5		3 vs. 4 0.012
CNLC							
Ⅰ	582	34.8	93.3	79.9	69.3	< 0.001	
Ⅱ	352	14.7	84.3	51.7	3		Ⅰ vs. Ⅱ < 0.001
Ⅲ	522	7.9	64.2	34.1	24.5		Ⅱ vs. Ⅲ < 0.001
Ⅳ	39	7.3	30.2	23.5	23.5		Ⅲ vs. Ⅳ 0.051
JIS							
0	75	44.8	100.0	89.7	85.3	< 0.001	
1	415	34.4	92.6	79.2	67.7		0 vs. 1 < 0.001
2	473	17.0	82.3	53.6	44.8		1 vs. 2 < 0.001
3	378	8.4	59.7	28.0	16.3		2 vs. 3 < 0.001
4	154	5.0	33.9	7.7	7.7		3 vs. 4 < 0.001
CLIP							
0	281	40.6	97.0	86.6	77.1	< 0.001	
1	445	22.3	89.9	68.6	57.0		0 vs. 1 < 0.001
2	329	13.1	73.9	42.3	35.2		1 vs. 2 0.004
3	254	10.0	62.5	31.8	17.9		2 vs. 3 < 0.001
4	141	6.0	41.6	20.3	20.3		3 vs. 4 0.012
5	45	6.1	17.3	/	/		4 vs. 5 < 0.001
CUPI							
1	1027	23.2	86.4	66.2	55.9	< 0.001	
2	421	8.2	57.7	30.9	26.6		1 vs. 2 < 0.001
3	47	4.7	29.7	19.5	9.7		2 vs. 3 < 0.001
Okuda							
I	798	24.9	88.6	70.7	61.3	< 0.001	
II	657	10.7	66.2	39.3	30.2		I vs. II < 0.001
Ⅲ	40	6.2	21.5	8.6	/		II vs. III < 0.001
GRETCH							
A	474	24.4	90.5	74.2	62.8		
B	981	13	72.8	49.0	41.3		A vs. B < 0.001
C	40	4.2	28.1	7.5	/		B vs. C < 0.001
Tokyo							
0	53	55.6	100.0	98.0	91.7		
1	246	34.0	96.2	82.5	67.4		0 vs. 1 < 0.001
2	389	23.2	86.6	63.9	56.1		1 vs. 2 0.003
3	321	13.8	73.3	52.5	46.1		2 vs. 3 < 0.001
≥ 4	486	7.8	57.1	25.0	15.0		3 vs.≥4 < 0.001

**Abbreviations**: HKLC, Hong Kong Liver Cancer; BCLC, Barcelona Clinic Liver Cancer; CLIP, Cancer of Liver Italian Program; JIS, Japan Integrated Staging score; LHR, Likelihood Ratio; CNLC, China liver cancer staging; CUPI, Chinese University Prognostic Index; GRETCH, Groupe d’Etude et de Traitement du Carcinome Hépatocellulaire.

### Predictive accuracy of the nine survival staging systems

As shown in Table 4, HKLC exhibited the lowest AIC value among the entire cohort, indicating superior predictive accuracy compared to other staging systems. Subsequent tests, including the linear trend χ² test and likelihood ratio test, demonstrated that the CLIP and the JIS systems exhibited strong performance in homogeneity (LHR χ²: 117.58) and gradient monotonicity (linear trend χ²: 411.00), respectively. Subgroup analyses showed that in the subgroups of HCV-infected individuals and those who had received radical therapy, CLIP had the most accurate predictive ability.

**Table 4:** Comparison of the discriminatory power, gradient monotonicity, and homogeneity of the nine staging systems in predicting survival after diagnosis of hepatocellular carcinoma in the overall population and in subgroups.

Staging system	Linear trend χ² test	LHR χ² test	AIC
HKLC	97.45	344.19	6942.88
CLIP	117.58	337.10	6949.46
JIS	79.63	411.00	6954.58
BCLC	116.45	324.81	6962.25
CNLC	85.64	318.83	6968.24
Tokyo	74.14	251.93	7035.13
Okuda	63.54	63.54	7119.51
CUPI	41.63	160.20	7126.82
GRETCH	41.77	41.76	7188.40
HBV-Infection Subgroup *N* = 1144 (76.5%)			
HKLC	100.13	307.06	4914.26
JIS	80.26	294.44	4926.92
CLIP	99.78	280.38	4940.94
CNLC	83.56	274.44	4946.88
BCLC	97.83	269.49	4951.83
Tokyo	67.85	218.88	5002.44
Okuda	59.69	150.05	5071.27
CUPI	42.24	145.29	5076.03
GRETCH	34.93	83.98	5137.34
HCV- Infection subgroup *N* = 58 (3.9%)			
CLIP	1.52	6.73	129.32
Okuda	1.20	6.15	129.897
CNLC	0.59	5.45	130.59
JIS	7.09	5.05	131.00
Tokyo	2.77	4.98	131.07
BCLC	3.74	3.54	132.51
HKLC	0.79	3.26	132.79
CUPI	0.51	1.61	134.44
GRETCH	0.03	0.90	135.15
Non-curative treatment subgroup *N* = 849 (56.8%)			
HKLC	32.85	136.37	3874.98
CLIP	51.80	134.87	3876.48
BCLC	39.80	124.07	3887.28
CNLC	32.21	121.47	3889.88
JIS	23.27	119.81	3891.54
Tokyo	25.09	86.59	3924.76
CUPI	19.71	80.82	3930.53
GRETCH	29.06	60.89	3950.46
Okuda	19.52	46.93	3964.42
Curative-treatment subgroup *N* = 646 (43.2%)			
CLIP	66.47	96.18	1867.03
BCLC	69.24	87.93	1871.28
HKLC	73.74	85.31	1873.91
JIS	62.42	78.39	1880.82
CNLC	55.24	73.37	1885.85
Okuda	47.29	67.34	1891.97
Tokyo	46.24	51.90	1907.31
CUPI	15.15	19.54	1939.67
GRETCH	11.13	17.04	1942.17

**Abbreviations**: AIC, Akaike information criterion; HKLC, Hong Kong Liver Cancer; BCLC, Barcelona Clinic Liver Cancer; CLIP, Cancer of Liver Italian Program; JIS, Japan Integrated Staging score; LHR, Likelihood Ratio; CNLC, China liver cancer staging; CUPI, Chinese University Prognostic Index; GRETCH, Groupe d’Etude et de Traitement du Carcinome Hépatocellulaire; HBV, viral hepatitis type B; HCV, viral hepatitis type C.

**Fig. 3:**
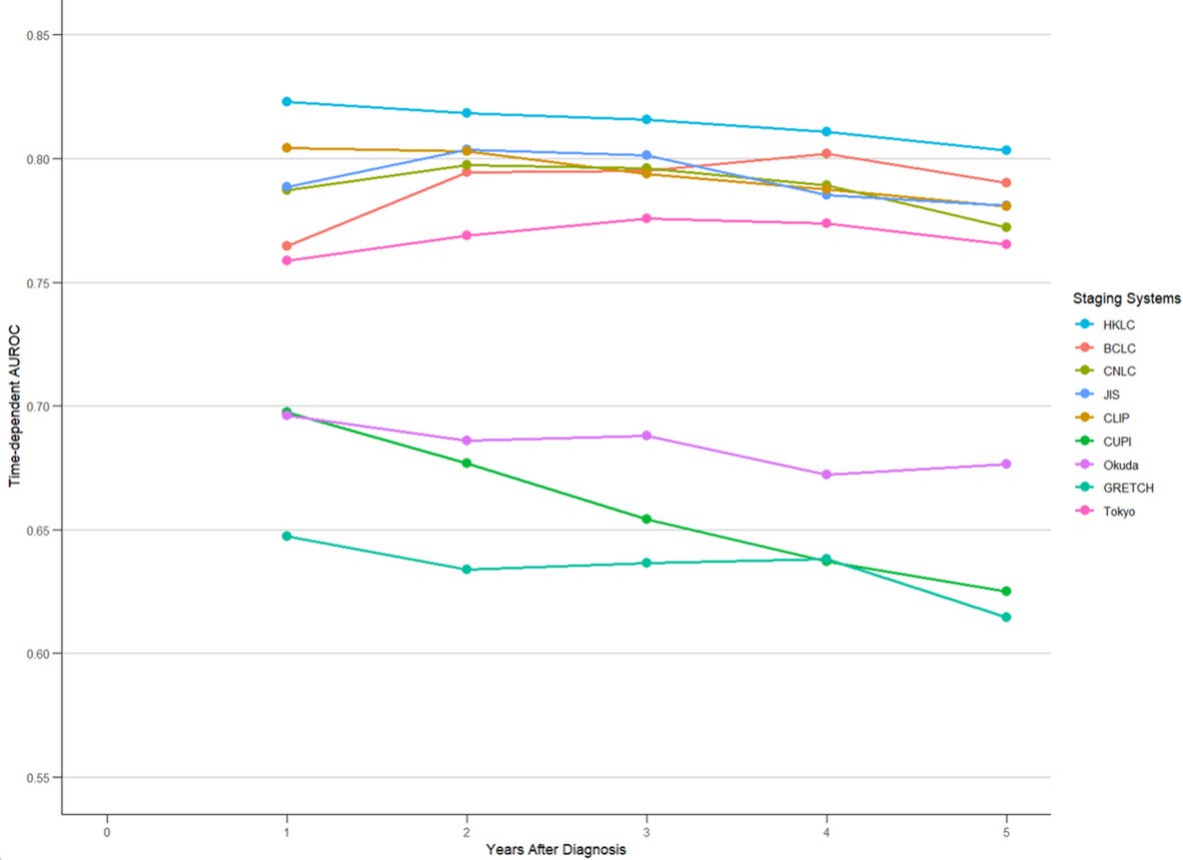
Comparisons of time-dependent area under the receiver operating characteristic curve (AUROC) of the nine staging systems for predicting mortality at each of the first 5 years after diagnosis.

Table 5 presents the AUC values and their 95% CIs for 1-, 3-, and 5-year mortality predictions. The HKLC exhibited AUC values of 0.823, 0.816, and 0.803 at the 1-, 3-, and 5-year time points, respectively, reflecting the highest discriminatory power among the staging systems, though these differences were not statistically significant compared to some other staging systems. Time-dependent ROC analyses demonstrated that the prognostic capacity of most staging systems progressively decreased over time; however, as shown in Fig. 3, HKLC consistently obtained the best AUC values at all time points.

**Table 5:** Comparisons of discriminatory ability evaluated by the area under the receiver operating characteristic curve (AUROC) for 1-, 3-, and 5-year mortality after diagnosis of hepatocellular carcinoma among the nine staging systems.

Staging system	1-year mortality			3-year mortality			5-year mortality		
	AUROC	95% CI	*P*-value	AUROC	95% CI	*P*-value	AUROC	95% CI	*P*-value
HKLC	0.823	0.797–0.848	Ref.	0.816	0.788–0.844	Ref.	0.803	0.769–0.838	Ref.
CLIP	0.804	0.776–0.832	0.387	0.794	0.764–0.823	0.202	0.780	0.744–0.818	0.291
JIS	0.789	0.760–0.817	0.001	0.801	0.786–0.839	0.509	0.781	0.748–0.816	0.342
CNLC	0.787	0.758–0.816	0.001	0.796	0.767–0.825	0.267	0.772	0.738–0.806	0.096
Tokyo	0.773	74.42–80.24	< 0.001	0.781	0.752–0.812	0.008	0.769	0.732–0.805	0.061
BCLC	0.765	0.741–0.789	< 0.001	0.795	0.766–0.825	0.231	0.790	0.753–0.827	0.715
CUPI	0.697	0.665–0.729	< 0.001	0.654	0.657–0.682	< 0.001	0.625	0.594–0.656	< 0.001
Okuda	0.696	0.665–0.727	< 0.001	0.688	0.657–0.719	< 0.001	0.677	0.640–0.713	< 0.001
GRETCH	0.647	0.619–0.675	< 0.001	0.636	0.605–0.668	< 0.001	0.614	0.574–0.655	< 0.001

**Abbreviations**: AIC, Akaike information criterion; HKLC, Hong Kong Liver Cancer; BCLC, Barcelona Clinic Liver Cancer; CLIP, Cancer of Liver Italian Program; JIS, Japan Integrated Staging score; LHR, Likelihood Ratio; CNLC, China liver cancer staging; CUPI, Chinese University Prognostic Index; GRETCH, Groupe d’Etude et de Traitement du Carcinome Hépatocellulaire; LHR, Likelihood Ratio.; CI, confidence interval.

## Discussion

Choosing the most appropriate staging system for HCC is critical for accurately predicting prognosis, developing personalized treatment plans, and optimizing healthcare resource utilization. However, significant controversy remains in China regarding which staging system offers the best predictive capability. In recent years, new staging systems incorporating artificial intelligence have been proposed, yet traditional staging systems continue to dominate clinical practice. This study selected nine commonly used clinical staging systems and analyzed a large multicenter dataset of 1,495 patients to identify independent risk factors associated with prognosis and compare the predictive accuracy of these systems.

In our study, 76.5% of cases were associated with HBV infection; however, HBV did not demonstrate a significantly higher risk of disease progression or mortality, likely due to the emphasis on antiviral treatment in Chinese clinical practice^26^. The study identified tumor burden, liver functional reserve, and the patient’s physical condition as independent risk factors. Among all the staging systems, the HKLC system demonstrated the highest predictive accuracy in the overall population. In the subgroups of patients receiving curative treatment and those infected with HCV, the CLIP system exhibited superior performance.

Tumor burden, liver functional reserve, and the patient’s physical condition are recognized as independent risk factors influencing tumor prognosis. Even with a relatively low tumor burden, poor liver function often results in unfavorable outcomes^27^. Therefore, maintaining adequate liver function reserve to meet physiological demands is critical for patients with HCC^27^. The Child-Pugh score is the most commonly used clinical indicator for evaluating liver function^28^. It includes all liver function-related independent risk factors identified in our study and is utilized in various staging systems to assess liver function^28,29^. Furthermore, as with other solid tumors, tumor burden remains a key factor influencing prognosis^1^. In our study, tumor size, number of tumor nodules, vascular invasion, and distant metastasis were identified as independent risk factors. For tumor size classification, different staging systems use different thresholds, such as tumors ˃5 cm or tumors involving more than 50% of the liver^30^. In this study, we adopted the maximum tumor diameter as the threshold, which is easier to assess. However, with the continuous advancement of imaging and molecular diagnostic technologies, an increasing number of early-stage HCC cases are being identified, necessitating further optimization of tumor size and burden classification standards^31,32^. Notably, recent evidence highlighted that preoperative imaging features, such as macroscopic tumor compression of the hepatic or portal vein, are independent predictors of microscopic biological invasiveness, including microvascular invasion and satellite nodules^33^. Integrating these early perivascular imaging markers into staging systems bridges the gap between macroscopic evaluation and microscopic pathology, significantly enhancing prognostic stratification and providing more accurate guidance for determining optimal surgical resection margins. Additionally, treatment modalities were identified as independent risk factors influencing prognosis.

Among all staging systems, survival trends showed a decline from early to advanced stages, as demonstrated by Kaplan–Meier survival curves. Additionally, all staging systems provided prognostic stratification for early-stage patients. For advanced-stage patients, most systems also exhibited good discriminatory ability, except for HKLC (Stage IV and V) and CNLC (Stage III and IV). In the overall population, HKLC demonstrated the best predictive accuracy, while CLIP and JIS showed the best monotonicity and homogeneity, respectively. In subgroup analysis, CLIP performed best in the HCV-infected subgroup and among patients receiving curative treatment, while HKLC demonstrated superior performance in the HBV-infected subgroup and among patients receiving non-curative treatments.

The biological behavior and progression of HCC are closely related to its underlying etiology^34^. Our study observed that the most effective staging system varied depending on the underlying etiology and treatment modality. The HKLC system, developed for the HBV-infected population, exhibited the highest predictive accuracy in the HBV-prevalent Chinese population^35^. In contrast, the CLIP system, based on a cohort predominantly infected with HCV, showed the best performance in the HCV-infected subgroup, which was expected^30^. The HKLC score includes performance status, Child-Pugh score, tumor burden (based on the Milan criteria), intrahepatic and extrahepatic vascular invasion, or metastasis, all of which are weighted in real-world populations, making its superior predictive accuracy reasonable^35^. However, the complexity of the HKLC system limits its clinical applicability, and it has not demonstrated the best predictive ability in HCV-prevalent Western countries^36,37^. The CLIP score, based on a retrospective cohort study predominantly composed of HCV-infected patients, has been validated in external cohorts in Canada, Italy, and Japan^9,30^. In our study, CLIP also demonstrated good predictive accuracy but did not account for the clinical status of patients. It is more suited to radical treatments such as percutaneous ablation, surgery, or liver transplantation. In particular, for the subgroup of patients undergoing curative surgery, the specific surgical approach and vascular management strategies are key determinants of clinical outcomes that are often not fully captured by traditional staging systems. For example, recent evidence demonstrates that while complex vascular procedures—such as middle hepatic vein resection during hemihepatectomy—may increase intraoperative blood loss and transient postoperative liver enzyme elevations, they do not significantly compromise long-term overall or recurrence-free survival. This highlights that aggressive surgical vascular management, when anatomically necessary for complete tumor clearance, remains oncologically safe and feasible. Therefore, comprehensively considering these detailed surgical interventions and vascular strategies in conjunction with established staging systems can provide a more accurate and clinically convincing prognostic evaluation for surgical patients^38^.

In addition to HKLC and CLIP, BCLC and CNLC demonstrated good predictive accuracy in our cohort. As the most widely used staging system globally, BCLC incorporates liver function, tumor burden, and patient performance status, providing recommended treatment options based on disease stage. Currently, BCLC is included in liver cancer treatment guidelines in Europe and the United States^10,39^. CNLC, proposed in 2017 and incorporated into the Chinese liver cancer treatment guidelines in 2017, similarly offers treatment recommendations based on staging^14^. Compared to BCLC, CNLC recommends more aggressive treatment options, placing more intermediate-stage patients in the radical treatment group, making it better suited to clinical practice in China^14^. Regional differences in treatment preferences significantly impact adherence to treatment recommendations based on staging systems and their influence on patient prognosis.

This study has some limitations. First, despite collecting data from 17 major hospitals in China, as a nationwide multicenter study, our findings may not fully represent the broader population of Chinese patients with HCC. Second, as this study was restricted to a Chinese population without external validation, caution should be taken when generalizing the findings to other East Asian or Western populations, given differences in liver disease etiology and treatment strategies. Third, although we compared the predictive performance of various HCC staging systems, the prognostic impact of treatment strategies recommended by these systems was not further evaluated. Additionally, as this was a multicenter retrospective study, potential inter-center heterogeneity in treatment preferences was not formally quantified. While treatment decisions across all centers were generally aligned with contemporaneous CNLC and BCLC guidelines throughout the study period, the potential influence of center-specific treatment practices on patient prognosis cannot be completely ruled out.

Since CNLC was first included in the Chinese liver cancer clinical guidelines in 2017, this study is the first to compare the predictive abilities of multiple staging systems, including CNLC, in the overall Chinese population. Furthermore, this study explored the performance of staging systems in subgroup populations. Most previous studies were single-center studies, typically limited to patient populations receiving specific treatments. In summary, our study suggests that HKLC demonstrates the highest predictive accuracy for HCC prognosis in China. Considering the etiology and treatment modality, the CLIP staging system shows superior predictive accuracy in HCV-infected patients and those receiving curative treatment. We look forward to more multicenter, prospective studies in the future to further validate and refine these staging systems, providing stronger theoretical and data support for the precise diagnosis and treatment of HCC.

## Data Availability

The original contributions presented in this study are included in the article/supplementary material. Further inquiries can be directed to the corresponding author.

## Abbreviations

AASLD: American Association for the Study of Liver Diseases
AFP: alpha-fetoprotein
AIC: Akaike information criterion
AST: Aspartate aminotransferase
AUROC: Area under the receiver operating characteristic curve
HKLC: Hong Kong Liver Cancer staging system
BCLC: Barcelona Clinic Liver Cancer
CI: Confidence interval
CLIP: Cancer of Liver Italian Program
CNLC: China liver cancer staging
CPS: Child-Pugh score
CT: Computed tomography
CUPI: Chinese University Prognostic Index
ECOG: Eastern Cooperative Oncology Group
EASL: European Association for the Study of the Liver
GRETCH: Groupe d’Etude et de Traitement du Carcinome Hépatocellulaire
HBV: Hepatitis B virus
HCC: Hepatocellular carcinoma
HCV: Hepatitis C virus
HR: Hazard Ratio
JIS: Japan Integrated Staging score
LHR: Likelihood ratio
MELD: Model for End-Stage Liver Disease score
MRI: Magnetic resonance imaging
TACE: Transarterial chemoembolization
